# Understanding the Barriers to Accessing Symptom-Specific Cognitive Behavior Therapy (CBT) for Distressing Voices: Reflecting on and Extending the Lessons Learnt From the CBT for Psychosis Literature

**DOI:** 10.3389/fpsyg.2018.00727

**Published:** 2018-05-15

**Authors:** Cassie M. Hazell, Kathryn Greenwood, Sarah Fielding-Smith, Aikaterini Rammou, Leanne Bogen-Johnston, Clio Berry, Anna-Marie Jones, Mark Hayward

**Affiliations:** ^1^Department of Primary Care and Public Health, Brighton and Sussex Medical School, University of Sussex, Brighton, United Kingdom; ^2^School of Psychology, University of Sussex, Brighton, United Kingdom; ^3^Research and Development Department, Sussex Partnership NHS Foundation Trust, Worthing, United Kingdom; ^4^Department of Neuroscience, Brighton and Sussex Medical School, University of Sussex, Brighton, United Kingdom

**Keywords:** cognitive behavior therapy, CBT, psychosis, barrier, access, hearing voices

## Abstract

The experience of hearing voices (‘auditory hallucinations’) can cause significant distress and disruption to quality of life for people with a psychosis diagnosis. Psychological therapy in the form of cognitive behavior therapy (CBT) for psychosis is recommended for the treatment of positive symptoms, including distressing voices, but is rarely available to patients in the United Kingdom. CBT for psychosis has recently evolved with the development of symptom-specific therapies that focus upon only one symptom of psychosis at a time. Preliminary findings from randomized controlled trials suggest that these symptom-specific therapies can be more effective for distressing voices than the use of broad CBT protocols, and have the potential to target voices trans-diagnostically. Whilst this literature is evolving, consideration must be given to the potential for a symptom-specific approach to overcome some of the barriers to delivery of evidence-based psychological therapies within clinical services. These barriers are discussed in relation to the United Kingdom mental health services, and we offer suggestions for future research to enhance our understanding of these barriers.

## Introduction

Hearing the voice of someone or something that is not physically present is a common experience that can cause distress and disruption to quality of life. Distressing voices are a core symptom of schizophrenia and other psychosis-spectrum disorders (World Health Organization [WHO], 1992; American Psychiatric Association, 2013). For the 220,000 people currently living with these diagnoses in the United Kingdom (National Institute for Health and Care Excellence [NICE], 2014a), the National Institute for Health and Care Excellence (NICE) recommends the provision of cognitive behavior therapy for psychosis (CBTp) as an adjunct to antipsychotic medication (National Institute for Health and Care Excellence [NICE], 2014b). However, CBTp has been criticized with respect to both its effect sizes and availability. In terms of effect sizes, meta-analyses confirm a small-moderate effect of CBTp on general positive symptoms (Wykes et al., 2007; Jauhar et al., 2014), and voices specifically where they are measured as a secondary outcome (Van der Gaag et al., 2014). With respect to availability, only 10% of patients with a psychosis-spectrum diagnosis have access to CBTp in the United Kingdom National Health Service (NHS) (Schizophrenia Commission, 2012; Haddock et al., 2014).

Cognitive behavior therapy for psychosis is a formulation-driven, broad therapy approach that provides the scope to address any mental health symptom that is problematic for the patient. By contrast, symptom-specific therapies target a single, pre-defined symptom. Commentators have suggested the effects of CBTp on psychosis symptoms may be enhanced by taking a symptom-specific approach, e.g., CBT that specifically targets distressing voices (CBTv) (Thomas et al., 2014; Lincoln and Peters, 2018); as opposed to distressing voices being one of a number of symptoms that may be targeted using broad CBTp protocols. CBTv assumes that it is not the experience of hearing voices in itself that causes distress. Instead, voice-related distress is maintained by endorsement of a specific set of beliefs in relation to voices (Birchwood and Chadwick, 1997). Eight randomized controlled trials (RCTs) have used this targeted approach to date, drawing upon a range of therapeutic techniques and frameworks that target the specific mechanisms maintaining voice-related distress, e.g., negative beliefs about the self, beliefs about voice omnipotence, and patterns of submissive relating. These studies reported effect sizes in the small-moderate range on at least one of the targeted outcomes, with several in the large range [see Lincoln and Peters (2018) for a review]. It is possible that CBT targeted at distressing voices might offer greater benefits to patients, but more definitive trials are needed to verify this.

If symptom-specific CBT can generate greater benefits for patients hearing distressing voices, we must consider the barriers to accessing this therapy within the NHS. A systematic review of barriers to the delivery of CBTp (Ince et al., 2015) found there to be two main obstacles to access: (1) limited resources, and (2) ambivalent attitudes of staff and patients. This paper will consider the extent to which symptom-specific CBT for voices (hereafter referred to as CBTv) can overcome the known barriers to accessing CBTp; and whether any additional barriers are generated by CBTv.

## Existing Barriers to Implementation of CBTp

### Limited Resources

Cognitive behavior therapy for psychosis is resource intensive as it is recommended for delivery over at least 16 sessions and facilitated by expert therapists. NICE recommend research into two issues that could reduce the resources required to deliver CBTp, and thereby increase its availability: (1) the duration of CBTp; and, (2) the ability of “briefly trained therapists” to deliver CBTp. Regarding the first recommendation, our meta-analysis of 10 controlled studies indicated that CBTp delivered over less than 16 sessions is effective in reducing psychosis symptoms (Hazell et al., 2016). These brief forms of CBTp were delivered exclusively by expert therapists (typically Clinical Psychologists). The second recommendation has been addressed by only two large-scale RCTs that have evaluated the outcomes of brief CBTp delivered by non-expert therapists, i.e., frontline practitioners (mental health practitioners without a formal therapy qualification) in receipt of brief training. Neither study reported a significant impact upon the positive symptoms of paranoid delusions or distressing voices (Turkington et al., 2006; Guo et al., 2017). A recently completed pilot RCT of brief CBT delivered to psychosis patients by non-expert therapists also reported small standardized between-group effect sizes on measures of hallucinations and delusions (Waller et al., 2018).

The evidence above suggests that brief CBTp can be effective, if therapy is delivered by expert therapists, and this brevity could enable more patients to be seen using the same resources. However, the limited availability of expert therapists would still leave many patients with no access to CBTp, and initiatives to increase the availability of expert therapists (Jolley et al., 2015) have had limited impact nationally. CBTv can also be delivered in brief forms. However, compared to CBTp which requires expert formulation, the targeted, mechanism-focused and manualized approach of some CBTv interventions may better lend itself to delivery by non-expert therapists. However, trials have yet to evaluate the delivery of CBTv by non-expert therapists, and research is required in this respect.

If CBTv was found to be effective when delivered by non-expert therapists following brief training, the demand on resources may remain unchanged as the majority of patients distressed by hearing voices with a psychosis diagnosis will also experience delusions (Mancuso et al., 2014). Given the overlap in cognitive-behavioral explanatory models for voices and delusions (Garety et al., 2001), it is possible that for some patients, an intervention for voices would reduce the need for further work targeting delusions. However, if a patient experiencing distressing voices and delusions were offered a brief, targeted intervention for each symptom then this would require a total number of sessions that is similar to the current recommendation of 16 sessions. For example, a patient could be offered eight sessions of guided self-help CBTv (Hazell et al., 2017a), plus six sessions of a worry intervention in the context of persecutory delusions (Freeman et al., 2015); 14 therapy sessions overall. A combined pathway of targeted therapies may not be less resource-intensive in terms of the number of sessions, but could be more widely available if it were found to be effective and deliverable by a larger and more cost-effective workforce of non-expert therapists.

### Ambivalent Attitudes of Patients and Clinicians

Clinicians, such as mental health nurses, psychiatrists, and psychologists, provide the gateway for patients to access psychological interventions for psychosis; and so understandably, when clinicians hold ambivalent attitudes regarding who is appropriate for CBTp, who will accept it and who will benefit, this can impact on both referrals and uptake (Prytys et al., 2011; Ince et al., 2015). Indeed, theory of planned behavior requires that implementation is desirable, associated with positive attitudes and perceived to be within behavioral control (Ajzen, 1991). Furthermore, patients’ ambivalent attitudes toward health, illness, and treatment also impact upon uptake of CBTp, with only 20–40% of patients taking up CBT when this is offered (Freeman et al., 2013). The knowledge and attitudes of clinicians and patients is thus argued to be critical to the implementation of psychological interventions.

The ambivalent attitudes of clinicians and patients are partly fueled by a lack of clarity regarding the focus of a broad CBT approach. Clinicians are uncertain about whether it is past, present, or future oriented; focused on psychosis or other symptoms; and how it works (Greenwood, 2017). In part, this is because broad CBT is collaborative and formulation-driven, with the focus arising out of shared goals, once therapy has started. This has the advantage of being flexible to meet a variety of patient needs but also means that the nature and target of CBTp is nebulous and difficult for clinicians and patients to grasp pre-therapy. One advantage of CBTv is that the focus is more transparent from the outset. If the intervention is also brief and manualized then the process, duration and expected outcomes will be clear. Both clinician and patient can then make informed choices about referring to or taking up CBTv.

Ongoing work from the second author (KG) has revealed that a further focus of clinicians’ ambivalent attitudes is psychosis experiences themselves. Patients with psychosis are perceived as unwilling or unable to take up CBTp; too distressed by their experiences, including voices; with CBTp being seen as a poor match for their needs. Clinicians are doubtful of their own ability to manage the difficult beliefs and emotions raised in their patients and are skeptical that CBTp can help with disturbing experiences. Psychosis patients have been seen as too symptomatic, with too little insight to undertake CBTp (Prytys et al., 2011). These perspectives impact in turn on patients, who believe they need to be strong and resilient before trying CBTp, rather than considering CBTp as a means to enhance their strength and resilience. A CBTv intervention that focuses on strengthening positive beliefs about the self (e.g., Hayward et al., 2016), and is valued by patients, with good outcomes has the potential to address many of these concerns, by demonstrating that patients can engage and respond well, even when the focus is on distressing voice-hearing experiences.

Waiting lists pose a further barrier to referral, as clinicians feel forced to make decisions about who is most appropriate or will benefit most from CBTp. This has often meant that those with most need, who have high levels of symptoms and distress, are the least likely to be referred. Whilst we would advocate that referrals should be a joint decision between clinician and patient (Greenwood, 2017), a brief focused intervention may address concerns about waiting lists, as more patients can be seen in less time.

Finally, ongoing work by the second author (KG) is revealing that patients themselves are less likely to take-up CBTp if it is viewed as too superficial when their experiences are spiritual, or too challenging when their experiences are perceived as real. There is evidence to suggest that patients may be more open to CBT if they believe their difficulties are, at least partially, psychological, and their outcomes may be better if they believe in the intervention and in their own ability to change (Freeman et al., 2013). Patients may therefore be reassured by interventions such as CBTv that focus specifically on the power of the voices and the distress caused, as opposed to the origin or meaning of their experiences.

## Does a Symptom-Specific Approach Generate Additional Barriers?

### The Need for Disclosure of Distressing Voices

Access to CBTp does not require a patient to disclose any distressing voices they may be experiencing as they could access therapy by virtue of their diagnosis and/or other symptoms Access to CBTv will require distressing voices to be disclosed and assessed prior to the commencement of therapy. However, many patients are reluctant to disclose their voice hearing experiences. At the onset of voice hearing, patients may not disclose because they are confused about the experience (Boyd and Gumley, 2007), in denial (Compton et al., 2008) or hope the voices will go away (Boydell et al., 2006). Furthermore, the voice content itself can act to prevent disclosure (i.e., voices telling the patient not to say anything) (Beavan and Read, 2010). Most often, people do not disclose their voice hearing experiences because of concerns about stigmatizing responses from family, friends, and the wider society (Beavan et al., 2011; Mawson et al., 2011; Hazell et al., 2017b; Bogen-Johnston et al., 2017). Even within mental health services, patients are concerned about the potential for stigma from clinicians (Beavan et al., 2011), and the fear of stigma is likely to be reinforced by clinicians who feel it is inappropriate to encourage and initiate conversations about hearing voices (Coffey and Hewitt, 2008). It is possible that by raising the awareness and availability of CBTv this will encourage clinicians to proactively talk to their patients about hearing voices, and thus create more opportunities for disclosure. However, any impact on disclosure is likely to be limited in the absence of a societal-level reduction in mental health stigma.

### Voice Hearing Across Diagnoses

It could be argued that a further implication of the adoption of a symptom-specific approach is concern around equity of access to treatment for all patients distressed by voice hearing experiences.

It is now well established that distressing voice hearing experiences are not confined to patients with a diagnosed psychosis-spectrum disorder. Studies comparing voice hearing experiences between patients with schizophrenia and patients with at least one other diagnostic class (recently reviewed by Waters and Fernyhough, 2017) has indicated that these experiences may share more similarities than differences. These findings suggest that there is likely to be significant unmet clinical demand from voice hearers with non-psychosis diagnoses. However, the evidence-base for psychological intervention such as CBTp is currently largely restricted to patients with a psychosis-spectrum disorder (Turner et al., 2014), hindering the translation of these approaches into other clinical populations.

Despite this paucity of evidence from RCTs, there are reasons to believe that voice hearers with non-psychosis diagnoses may well benefit from CBT-based interventions. Whilst comparative studies have typically been small-scale, and are currently few in number, they have strongly hinted toward the presence of shared trans-diagnostic cognitive-behavioral mechanisms for voice hearing. For example, voice hearers with BPD (Hepworth et al., 2013) or bipolar disorder (Hammersley et al., 2010) report similar beliefs about their voices to those with a diagnosed psychosis-spectrum disorder. This preliminary evidence raises the possibility that CBT-based interventions evaluated in the context of psychosis-spectrum disorders may be suitable for other populations, were these to be made routinely available.

Of course, aside from the practical implications for services already struggling to meet the significant demand for CBTp, this suggestion is prefaced with a number of important caveats. First, we need to verify whether the mechanisms that maintain voice-related distress in those with a psychosis diagnosis are the same for those who hear voices with a non-psychosis diagnosis. Second, there is a need for formal empirical evaluation of the transdiagnostic potential of symptom-specific CBT-based approaches before this approach can be recommended without reservation. Third, it remains unclear whether the voice hearing experiences of patients with non-psychosis diagnoses should be considered a priority for treatment, over and above the ‘core’ diagnostic features associated with their diagnosis. This can be broken down into two elements; (i) to what degree would voice hearers without a psychosis-spectrum diagnosis choose to engage with voice-focused interventions, given the alternative of access to therapies targeting other disorder-relevant etiological processes; and (ii) are distressing voices best targeted by symptom-specific CBT-based approaches, or by interventions targeting features considered more primary to the particular non-psychosis diagnosis? It is possible, for example, that distressing voices experienced in the context of PTSD may respond better to trauma-focused interventions – a possibility that has yet to be tested empirically.

Regardless of the state of the evidence, steps can be taken to ensure that unnecessary barriers to treatment are removed. One particular barrier in the context of these populations is the fact that voice hearing typically receives only a fleeting mention in the diagnostic criteria for non-psychosis disorders, and as a result, these experiences may be overlooked by clinicians, or considered – rightly or wrongly – to be an accessory feature of a more primary presentation. There is a clear need for awareness-raising initiatives targeting clinicians working with populations in which voice hearing is commonly experienced. Voice hearing should be routinely and explicitly addressed during assessment across diagnoses in order to gauge the degree to which these experiences are seen as a treatment priority for the hearer. This process will facilitate clinical decision-making and access to appropriate services where they are available.

Despite our tentative support for the transdiagnostic approach to CBTv, we must offer an additional caveat for consideration. That is, between-diagnosis differences in service engagement may moderate the availability of CBTv. For example, people with a personality disorder (PD) diagnosis are more likely to seek help from services compared to people with other mental health diagnoses (Twomey et al., 2015). By contrast, psychosis-spectrum patients with a high level of symptoms typically have poor engagement with mental health services (Lecomte et al., 2008). Based on this evidence, we could predict that a disproportionate amount of the available CBTv resource might be given to those distressed by voices with a PD diagnosis compared to those with psychosis-spectrum diagnoses because they are more visible within mental health services. This is an issue that referring clinicians might benefit from keeping in mind.

## Discussion

A significant limitation of our paper is a consideration of CBTv only within the context of the NHS. This is largely due to the paucity of research on the issue of access to CBTp and CBTv outside of the United Kingdom. It is possible that some, if not all, of the barriers proposed here will be irrelevant in some non-UK mental health services; and that there may be additional barriers to access that we have not considered.

We have discussed these barriers in reference to generic CBTv. But, as mentioned previously, there are multiple mechanisms proven to maintain voice-related distress that could be conceptualized as therapy targets, i.e., negative beliefs about the self, beliefs about voice omnipotence, and patterns of submissive relating. The findings from our research suggest that it is possible to target and improve each of these mechanisms within a brief form of CBTv (Hazell et al., 2017a). Furthermore, our research also suggests that targeting coping strategies directly may improve voice-related distress and subjective recovery; with potential effects on mood and other voice phenomena (Hayward et al., 2018). However, these studies are preliminary; thus we are not yet able to verify whether these mechanisms mediate voice-related distress treatment outcomes, and whether the mediating pathways are additive or overlapping for these mechanisms. That is, we need to establish what mechanisms produce the greatest improvements in voice-related distress, and whether these mechanisms have differential effects on treatment outcomes. Future trials of CBTv should include measures related to the hypothesized therapy mechanisms to help us answer these questions. Our paper suggests that CBTv will not be a panacea for all of the barriers that limit access to psychological therapy for psychosis patients, but in terms of existing barriers to accessing CBTp, there is some scope for optimism. CBTv may: (1) have potential to reduce the resources required if brief forms of therapy can be effectively delivered by a large and cost-effective workforce of briefly trained therapists; and (2) address some of the concerns of clinicians and patients about the focus and likely outcomes of therapy. However, CBTv may generate further barriers as patients will be required to disclose their voice hearing experiences prior to the commencement of therapy, and additional demands on limited resources may be generated by a move toward offering therapy trans-diagnostically.

Further research is required to explore and understand more about the potential barriers to accessing CBTv. Specifically, this paper suggests that future research should focus upon:

(1) The ability of briefly trained therapists to effectively deliver the brief and manualized forms of CBTv;
(2) The attitudes of therapists, clinicians, and patients toward CBTv;
(3) The willingness of patients and clinicians to talk about voice hearing experiences;
(4) The extent to which CBTv is a treatment priority and can be effective for the treatment of distressing voices outside of a psychosis context;
(5) Understanding the barriers to accessing CBTv outside the United Kingdom.

CBTv does not yet have a sufficient evidence-base to warrant widespread implementation in mental health services. There is a need for more large-scale, high-quality studies exploring the effectiveness of these symptom-specific interventions. However, if we wait until this evidence-base has been established before considering these barriers to access then the issue of access is unlikely to be resolved. By proactively reviewing the barriers to CBTp, considering the additional barriers that may be generated by a symptom-specific approach, and formulating potential solutions, we hope to prevent this situation from occurring.

## Author Contributions

CH conceived the idea for this paper, with critical input from all other authors. CH and MH wrote the initial draft of this manuscript, with significant and critical edits from KG, SF-S, AR, CB, LB-J, and A-MJ. All authors have approved the final version of this manuscript.

## Conflict of Interest Statement

KG and MH are Clinical Psychologists with extensive experience of delivering CBTp and CBTv. MH is the Director of the Sussex Voices Clinic (a service that offers psychological therapies to people distressed by hearing voices) – both LB-J and A-MJ work within the clinic. KG is currently in receipt of funding to develop and evaluate a tool aimed at improving the uptake and implementation of CBT for psychosis. MH, CH, and KG are currently applying for funding to trial a brief CBTv intervention. The other authors declare that the research was conducted in the absence of any commercial or financial relationships that could be construed as a potential conflict of interest. The reviewer LV and handling Editor declared their shared affiliation.
